# Design of an algorithm for measuring psychosocial risk factors in higher education teachers

**DOI:** 10.47626/1679-4435-2025-1468

**Published:** 2025-11-04

**Authors:** Luisa Fernanda Becerra Ostos, Pedro Pablo Castañeda Ocampo, Janer Mauricio Guzmán Higuera

**Affiliations:** 1 Specialization Occupational Risk Management, Occupational Safety and Health, Corporación Universitaria Minuto de Dios, Bogotá, Colombia

**Keywords:** disease prevention, risk management, algorithms, mental health, faculty., prevención de enfermedades, gestión de riesgo, algoritmos, salud mental, docentes.

## Abstract

**Introduction:**

Psychosocial factors have represented a major challenge for occupational safety and health, being one of the main causes that affect individuals’ psychosocial well-being. Teachers are no exception, as they are exposed to multifactorial variables that impact their health.

**Objectives:**

To design an algorithm that measures the risk of exposure to psychosocial factors and guides the definition of specific control measures for higher education teachers.

**Methods:**

Specific formulas were developed to tabulate responses, assigning scores from 1 to 5 points according to each selected option. This approach made it possible to average the opinions provided by the evaluated teachers. The formulas were applied in a pilot test to determine the level of risk of exposure to psychosocial factors, in accordance with the previously established scale.

**Experience report:**

The designed formulas allowed for the summation and averaging the evaluated dimensions, generating a matrix with the individually obtained responses. The overall calculated risk level was low, with values (between 3.6 and 4.1), although specific dimensions, such as task demands and work changes, reached a medium level of risk (3.3), according to the established scale. Finally, intervention options aimed at managing these risks were described.

**Conclusions:**

The proposed algorithm represents a significant advance in the efficient management of psychosocial factors affecting teachers. This tool facilitates the analysis of risk levels and the planning of appropriate actions, providing a practical approach to address psychosocial challenges within this academic population.

## Abstract (es)

**Introducción:**

Los factores psicosociales han representado un desafío significativo para la seguridad y la salud en el trabajo, siendo una de las principales causas que afectan el bienestar psicosocial de las personas. Los docentes no son una excepción, pues se exponen a variables multifactoriales que impactan su salud.

**Objetivos:**

Diseñar un algoritmo que mida el riesgo por exposición a factores psicosociales y oriente la definición de medidas de control específicas para docentes de educación superior.

**Métodos:**

Se diseñaron fórmulas específicas para tabular las respuestas, asignando valores entre 1 y 5 puntos según cada respuesta, permitiendo así promediar los dictámenes emitidos por los docentes evaluados. Las fórmulas fueron aplicadas en una prueba piloto, para determinar el nivel de riesgo de exposición a factores psicosociales, según el baremo previamente establecido.

**Relato de experiencia:**

Las fórmulas desarrolladas permitieron sumar y promediar las dimensiones evaluadas, generando una matriz con las respuestas obtenidas individualmente. Se calculó un nivel de riesgo bajo ( entre 3,6 y 4,1); no obstante; no obstante, existen dimensiones específicas, como las demandas de la tarea y los cambios en el trabajo, que alcanzaron un nivel de riesgo medio, con valor de 3,3, de acuerdo con el baremo establecido. Finalmente, se describieron opciones de intervención para gestionar los riesgos.

**Conclusiones:**

El algoritmo propuesto constituye un avance significativo en la gestión eficiente de factores psicosociales que afectan a los docentes. Esta herramienta facilita el análisis de los niveles de riesgo y la planificación de acciones pertinentes, ofreciendo un enfoque práctico para abordar los desafíos psicosociales presentes en esta población docente.

## INTRODUCTION

Psychosocial risk factors comprise a variety of elements that that affect individuals’ biopsychosocial well-being and currently pose a major public health challenge, given their detrimental impact on health. In this context, exposure to stressful circumstances may impair adaptive capacity across diverse environments, leading to decreased quality of life and reduced functional performance.^1^

The International Labour Organization^2^ reports an estimated loss of 12 billion workdays attributable to anxiety and depression. Furthermore, the World Mental Health Report by the World Health Organization (WHO) indicates that in 2019 15% of the working age population experiences health conditions related to psychological violence, discrimination, and inequality, which adversely affect their mental health. The WHO^3^ estimates that 4.4% of the global population, equivalent to over 300 million people, exhibits symptoms of depression, making it the world’s leading cause of disability.

The higher education sector is not immune to this problem, since it bears responsibility for professional training and for the scientific and technological advancement of a nation. The teaching profession also involves occupational and professional risks due to the exposure to high emotional demands, psychosocial risk factors, extensive working hours, and excessive workloads, among others. Moreover, individual characteristics and the role each person play in the academic environment can contribute to mental fatigue and, in some cases, to work-related disorders such as mobbing and occupational stress.^4^ These issues have inspired important theoretical insights into teachers’ occupational health.

In recent years, an increase in the prevalence of mental disorders has been observed within educational institutions. The link between psychosocial risk factors and mental disorders is complex and multifactorial. Perales et al.^5^ found a significant correlation between these two variables, indicating that psychosocial risk factors may raise exposure to stress and suicidal ideation, thus contributing to possible mental health problems. Likewise, Lemos et al.^6^ observed an association between psychosocial risk factors and mental disorders among higher education teachers, showing that workers exposed to high levels of occupational stress exhibited a considerably higher risk of developing depression. The authors also highlighted that psychosocial risk factors such as job demands, workload, and the work-family interface are often associated with mental disorders like depression, anxiety, sleep disorders, substance use, and mood disorders.

These findings underscore the need to explore these factors, which are constantly evolving within the occupational environment. As demonstrated by Lara & Pando,^7^ the study of psychosocial risk factors has had a substantial impact in this field and, over the decades, research has focused on identifying and understanding how health-related manifestations induced by mobbing and occupational stress, acknowledging their impact on the mental health of educational workers. Pioneering studies in the area have emphasized the importance of developing effective detection methods, while the current era introduces new challenges and opportunities with the integration of technological tools into the process.

Hence, the development of practical and easily comprehensible tools is essential for analyzing the relationships arising from psychosocial risk factors and mental health. The creation of algorithms is particularly useful in this context; they are constructed through a finite sequence of instructions to address a specific type of problem, exhibiting characteristics such as finiteness, definitiveness, input, output, and effectiveness, while converging into a dynamic feedback that has a significant impact on the population involved.^8^ In this context, the creation of health-related algorithms has been extensively explored by several authors and experts in areas such as medical informatics, epidemiology, clinical research, and health care. Por example, Berenice^9^ investigates the challenges posed by the coronavirus disease 2019 (COVID-19) pandemic, highlighting the importance and timeliness of developing algorithms to identify and prevent the numerous risks faced by the population.

Regarding psychosocial risk factors in Colombia, the Ministry of Social Protection^10^ offers models such as a mental health care guide, a key resource for preventing mental disorders in the workplace that provides valuables tools to identify and assessed psychosocial risk factors, supporting the creation of algorithms that help detect mental health problems and currently contribute to address the consequences derived from psychosocial risk factors. Nevertheless, these strategies mainly target the general population. Despite the existing resources, there is still no concrete solution to the issue, due to limited integration of the components required to design a comprehensive care pathway for psychosocial factors specifically affecting the teaching staff.^11^ Therefore, the development of an algorithm providing direct solutions, with clear and concise responses.

Hence, the aim of this study was to design an algorithm to measure the risk of exposure to psychosocial risk factors and to guide the definition of control measures for higher education teachers. The development of this algorithm contributes to the assessment of teachers’ well-being, the quality of teaching, and the efficient management, while ensuring and promoting a culture of well-being within the educational environment.

The present study has practical relevance for higher education institutions, as the algorithm makes it possible to measure psychosocial risk factors and to produce tangible solutions that improve the quality of life of the study population and foster a culture of continuous improvement in working conditions. Moreover, occupational health and safety teams will have additional tools to support and expedite management processes for preventing psychosocial factors, thereby reducing potential mental health disorders among the teaching staff.

## METHODS

This study uses a quantitative approach with a descriptive scope. According to Ramos,^12^ this method can be applied when the characteristics of the phenomenon under study are known; furthermore, it allows the identification of data patterns and trends that can support algorithm development, including the selection of relevant variables, definition of relationships between them, and assessment of algorithm performance.

### POPULATION

The study population consisted of 20 higher education teachers from two institutions in Bogotá and Barrancabermeja, Colombia. A non-probabilistic sampling method was used. As noted by Pereyra & Vaira,^13^ this type of sampling can also be applied in quantitative research, being useful for collecting data from a specific population or in cases of limited resources. Participants were therefore selected by convenience.

### INSTRUMENT

This study used a database derived from two previous investigations conducted with higher education teachers from two Colombian institutions.^14^,^15^ Access to this database was granted through prior authorization from the participating institutions. An Excel-based template was designed to obtain the required quantitative results. Data were collected using a questionnaire developed by the research group at Corporación Universitaria Minuto de Dios to access psychosocial risk factors among higher education teachers. This questionnaire covers three main dimensions: occupational, non-occupational, and individual factors, following López framework,^16^ which highlights the importance of establishing effective processes to achieve the desired outcome from the available data.

According to Sánchez et al.,^17^ a rating scale is a standardized scoring system applied to an instrument based on researcher-defined criteria. In this study, the following scale was used to quantify risk levels among the target population: 0.0-≤ 1.8, very high risk; > 1.8-≤ 2.8, high risk; > 2.8-≤ 3.5, medium risk; > 3.5-≤ 4.3, low risk; > 4.3-≤ 5.0, no risk.

### PROCEDURE

This research included 20 higher education teachers from two Colombian universities. A self-developed instrument, previously validated for content, was administered to identify the psychosocial risk factors to which participants were exposed. After reviewing data from earlier studies, the formula designed for the development of the algorithm was applied. Additionally, the company UniMinas SAS provided and authorized the use of a matrix that was adapted into a customized risk validation tool containing formulas and rating scales for risk level assessment. This tool was then used to analyze the results and develop the algorithm based on the observed risk levels.

### ANALYSIS OF INFORMATION

The instrument for identifying psychosocial risk factors among higher education teachers enabled the tabulation of responses from the characterization questionnaire applied to participants. Each question was assigned a score from 1 to 5, producing a quantitative factor as determined for this study. The sum of the responses for each item allowed for the calculation of an average score for each assessed factor and dimension. Likewise, the “If” Excel formula was applied to define risk levels, using the established rating scale as a reference.

The category thresholds were defined using two criteria. First, median, which divides the sample into two equal parts; in this study, it was 2.8. Second, percentiles, which divide the sample into 100 equal parts; in this study, the 25th, 50th, 75th percentiles were used. The resulting thresholds were the following: 0.0-≤ 1.8, very high risk; > 1.8-≤ 2.8, high risk; > 2.8-≤ 3.5, medium risk; > 3.5-≤ 4.3, low risk; > 4.3-≤ 5.0, no risk. These thresholds can be adjusted, depending on the objectives of the measuring instrument and on the characteristics of the target population. For instance, to increase scale sensitivity, the thresholds for high, moderate, and low risk can be narrowed.

Subsequently, a consolidated report was prepared, summarizing the results of the surveyed group. This provided a comprehensive overview of risk levels and identified the possibility of planning preventive measures or, when necessary, implementing an epidemiological surveillance system as an intervention plan based on the identified needs.

### ETHICAL CONSIDERATIONS

This research follows the Deontological Code of Psychology, which defines ethical standards for addressing psychosocial risks, prioritizing the principles of beneficence, nonmaleficence, autonomy, justice, truthfulness, solidarity, loyalty, and fidelity, along with the applicable legal provisions.^18^ Furthermore, article 30 of this code outlines the psychologist’s responsibility to ensure the secure and confidential preservation of psychological data, interviews, and test results, regardless of the medium in which they are stored. Likewise, the values of honesty, transparency, and social responsibility, among others, are of utmost importance to comply with the Code of Ethics and Good Governance.^19^

## EXPERIENCE REPORT

Based on the previous data from higher education teachers, evaluated using a content-validated instrument to assess psychosocial risk factors, notable sociodemographic differences were observed. In the first group, 66% were men, while in the second, 87% were women. However, both groups shared similar life-stage characteristics: approximately 70% were between 36-46 years old and over 60% held a master’s degree. Regarding intra-labor psychosocial factors, the most affected dimension in this population, the first group experienced higher exposure to workload and long working hours, whereas the second group showed greater exposure to task demands, workplace changes, decision-making, and control factors.

Based on the previously mentioned data, tabulation and analysis were conducted using a matrix, applying the formulas required for algorithm structuring, along with the corresponding guidelines to obtain the intended output and determine the risk level of the surveyed population. From the results of this procedure, the tool provides two possible interventions plans tailored to the specific needs of each institution.

The formulas were created in Microsoft Excel and include build-in function for summing, averaging, and generating a rating scale to define levels of psychosocial risk. Formula 1 assigns a numerical value from 1 to 5, according to each respondent’s answer: always (5), almost always (4), sometimes (3), rarely (2), and never (1). Formula 2 computes the mean score for each evaluated dimension, based on the participants’ responses. Formula 3 generates a matrix representing individual and/or group evaluations. For this step, the evaluated domains and their respective scores are tabulated, and the result is divided by 10,000.

Formula 4, in turn, generates the corresponding intervention plan options for each evaluated dimension or factor, considering the obtained assessments, feedback, and any required training actions. Finally, formula 5 determines the risk level using the scores obtained for each dimension and the tabulated data from formula 3, in order to apply the previously constructed rating scale.

Table 1 presents the formulas used in the present study, which were created in Microsoft Excel and include built-in functions for summing and averaging risk levels. As noted by Gutiérrez et al.,^20^ these formulas can be applied in algorithm development and serve as rating scales that assign numerical values to individuals based on test or survey outcomes. They are particularly useful for assessing algorithm performance compared to a reference group.

**Table 1:** Formulas used in algorithm development

Formulas	Description of the formula
1: Assignment of a numerical value	“=SI.ERROR(SI(Z5=“Siempre”;5;SI(Z5=“Casi siempre”;4;SI(Z5=“Algunas veces”;3;SI(Z5=“Casi nunca”;2;SI(Z5=“Nunca”;1)))));”“)”
2: Mean score for each evaluated factor and dimension	“=PROMEDIO(DE5:DJ5)”
3: Matrix for individual and/or group assessment	“=BUSCARV($E$9;Tabulación!$A$4:$GX$9999;192;0)”
4: Intervention plan	“=BUSCARV(G17;’Plan de Intervención’!$D$10:$F$14;2;0)”
5: Calculation of risk level	“=SI(F16<=1,8;”Muy alto”;SI(Y(F16>1,8;F16<=2,8);”Alto”;SI(Y(F16>2,8;F16<=3,5);”Medio”;SI(Y(F16>3,5;F16<=4,3);”Bajo”;SI(Y(F16>4,3;F16<=5);”Sin riesgo”)))))”

Similarly, a rating scale for assessing risk levels was designed (Table 2). This table presents the levels of risk for psychosocial factors with a minimal margin of error, in order to obtain the most meaningful evaluation possible.^21^

**Table 2:** Rating scale for assessing risk levels

Score	Risk level
0.0-≤ 1.8	Very high
> 1.8-**≤** 2.8	High
> 2.8-≤ 3.5	Medium
> 3.5-≤ 4.3	Low
> 4.3-≤ 5.0	No risk

**Figure 1:**
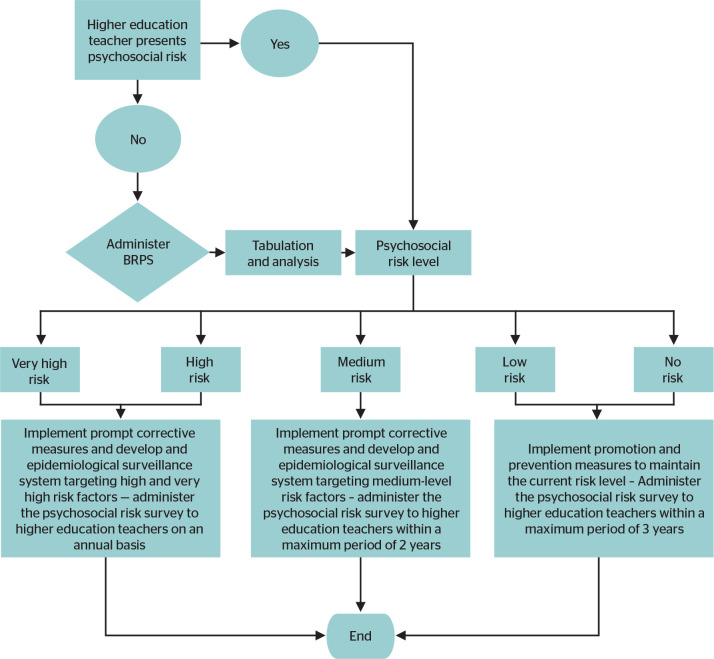
Algorithm for the assessment of psychosocial risks among higher education teachers.

Figure 1 shows the algorithm designed by the research team, which considers the risk levels (very high, high, medium, low, and no risk) and the respective actions to be implemented at each level. The algorithm supports the identification of teachers’ exposure to psychosocial risk and provides criteria for implementing disease prevention and health promotion measures. This is consistent with Gómez’s framework,^22^ which highlights that algorithms can help resolve conflicts and provide solutions to the identified problems.

BRPS = *Batería Riesgo Psicosocial* (Psychosocial Risk Battery).

For the sample to which the algorithm was applied, Table 3 indicates that the studied population shows a low risk level in the occupational, non-occupational, and individual dimensions. However, as an added value, and since algorithm itself does not include this component, the development of intervention plans tailored to each identified risk level in each case is proposed, thereby ensuring a customized and effective response to the detected needs. In line with the proposals of various authors, it is recommended to apply the algorithm in an abstract manner to stablish strategies and actions that, when properly implemented, are useful for the prevention of psychosocial risk factors.^23^

**Table 3:** Results from the application of the pilot test among higher education teachers and intervention plans

General report of findings Psychosocial risk factors among higher education teachers					
Factors	Dimensions	Score	Risk level	Intervention plan	
				Option 1	Option 2
Occupational	Relationship with supervisor/management style/power relationship	3.8	Low	Identify areas for improvement in the relationship and address them proactively.	Promote joint decision-making with the supervisor.
	Relationship with coworkers/work climate	3.8	Low	Identify and manage possible tensions or conflicts among coworkers.	Implement a mentoring program for new employees.
	Work environment conditions	3.9	Low	Improve the ergonomics of workspaces.	Implement labor flexibility policies (telework, flexible schedules).
	Job demand conditions	3.3	Medium	Review and adjust responsibilities and workload as needed.	Provide technological tools to optimize task efficiency.
	Workload	3.7	Low	Implement remote work or telecommuting policies.	Offer options of like part-time or job-sharing schedules.
	Decisions and control	3.8	Low	Provide training in effective decision-making.	Establish a feedback mechanism to assess decision quality.
	Work changes	3.4	Medium	Monitor the impact of changes and make adjustments as needed.	Offer emotional support and resources to cope with resistance to change.
	Training/professional development/orientation	3.8	Low	Conduct regular assessments of employees’ training needs.	Encourage mentoring and coaching programs to support professional growth.
	Acknowledgment and occupational well-being	3.9	Low	Administer satisfactions surveys to evaluate workplace well-being.	Provide flexible schedule options or remote workdays.
	Total for occupational factors	3.7	Low		
Non-occupational	Transportation and mobility	4.1	Low	Implement a corporate transportation system.	Facilitate access to bicycles or safe walking routes.
	Work-life balance	3.6	Low	Encourage wellness programs and extracurricular activities.	Provide support for stress management and mental health promotion.
	Total for non-occupational factors	3.8	Low		
Individual	Coping strategies/stress management/personality	3.7	Low	Coping: Hold regular workshops on stress management and relaxation techniques. Stress: Introduce flexible work policies to reduce work-related stress. Personality Establish programs for personal and professional development.	Coping: Foster open communication so employees can share their concerns. Stress: Provide self-help resources, such as meditation and breathing techniques. Personality: Offer personality self-assessment tools to support individual growth.
	Total for individual factors	3.7	Low		

Nevertheless, the evaluated higher education teachers to whom the algorithm was applied demonstrated a low psychosocial risk level. However, a medium-rated variable concerning work changes was identified. These changes reflect the transformations that higher education has undergone in recent years. This aligns with the study by García et al.,^24^ which highlight how social transformations and recent adjustments in labor reforms have increased psychological demands in higher education teachers, as they feel their opinions are ignored and their work has undergone sudden changes, resulting in less recognition of their role in society.

This algorithm was designed to measure risk levels among higher education teachers, based on other algorithms previously applied in different contexts, in order to facilitate data assimilation and informed decision-making.

## CONCLUSIONS AND RECOMMENDATIONS

The constructed algorithm allows for the conceptualization of psychosocial risk factors that arise when work demands exceed workers’ capacities and resources, leading to psychological overload that affects the population under study. Furthermore, two ways of interpreting these factors can be identified, through which higher education teachers visualize and determine possible measures to prevent the assessed factors, taking into account task execution, autonomy, and decision-making capacity, so as to improve and enhance their current work performance.^25^

It is worth noting that the algorithm was developed following recommendations from authors highlighting the need for using firsthand information to create new knowledge.^26^ it is also important to clarity that several existing algorithms from different specialties provided support and guidance for the construction of the present algorithm. The collected data enabled the design of an algorithm specific for higher education teachers and the formulation of intervention suggestions to help mitigate psychosocial risk factors.

This approach significantly contributes to promoting teachers’ mental health, while fostering a healthier work environment. It seeks to enhance continuous training processes and strengthen epidemiological surveillance systems, thereby positively impacting the quality of life and job satisfaction of this population.
